# Relevance of amyloid precursor-like protein 2 C-terminal fragments in pancreatic cancer cells

**DOI:** 10.3892/ijo.2012.1553

**Published:** 2012-07-13

**Authors:** HALEY L. PETERS, AMIT TULI, XIAOJIAN WANG, CUILING LIU, ZENGGANG PAN, MICHEL M. OUELLETTE, MICHAEL A. HOLLINGSWORTH, RICHARD G. MacDONALD, JOYCE C. SOLHEIM

**Affiliations:** 1 Eppley Institute for Research in Cancer and Allied Diseases;; 2 Departments of Biochemistry and Molecular Biology and; 3 Pathology and Microbiology, University of Nebraska Medical Center, Omaha, NE 68198, USA;; 4 Department of Pathology, Peking University Health Science Center, Beijing 100191, P.R. China;; 5 Department of Pathology, City of Hope National Medical Center, Duarte, CA 91010, USA

**Keywords:** amyloid precursor protein, amyloid precursor-like protein 2, β-site APP cleaving enzyme, pancreatic cancer

## Abstract

In some cellular systems, particularly neurons, amyloid precursor-like protein 2 (APLP2), and its highly homologous family member amyloid precursor protein (APP), have been linked to cellular growth. APLP2 and APP undergo regulated intramembrane proteolysis to produce C-terminal fragments. In this study, we found comprehensive expression of APLP2 C-terminal fragments in a panel of pancreatic cancer cell lines; however, APP C-terminal fragments were notably limited to the BxPC3 cell line. Extensive glycosaminoglycan modification on APLP2 was also found in the majority of pancreatic cancer cell lines. Glycosaminoglycan-modified and -unmodified APLP2, and particularly APLP2 C-terminal fragments, also demonstrated increased expression in oncogene-transformed pancreatic ductal cells. Additionally, elevated APLP2 levels were confirmed in human pancreatic cancer tissue. Downregulation of APLP2 and APP expression, alone or in combination, caused a decrease in the growth of a pancreatic cancer cell line with representatively low APP C-terminal fragment expression, the S2-013 cell line. Furthermore, we found that treatment with β-secretase inhibitors to block formation of APLP2 C-terminal fragments decreased the growth and viability of S2-013 cells, without affecting the survival of a non-transformed pancreatic ductal cell line. In conclusion, our studies demonstrate that abundant APLP2, but not APP, C-terminal fragment expression is conserved in pancreatic cancer cell lines; however, APP and APLP2 equally regulated the growth of S2-013 pancreatic cancer cells. Chiefly, our discoveries establish a role for APLP2 in the growth of pancreatic cancer cells and show that inhibitors preventing APLP2 cleavage reduce the viability of pancreatic cancer cells.

## Introduction

Amyloid precursor-like protein 2 (APLP2) is a protein that is well conserved between human and mouse, and it has high homology to another ubiquitously expressed family member, amyloid precursor protein or APP (1). Similarity between APLP2 and APP sequences is the primary explanation for a redundancy in some cellular functions; however, knock-out studies in mice have demonstrated an essential and unique function in viability for APLP2 that remains unidentified (2–5). As shown in various reports, APLP2 and APP are involved in cell migration, signaling, adhesion, proliferation and healing (1,4,6–12). As demonstrated by our laboratory, APLP2 can also bind to major histocompatibility complex class I molecules (receptors that present tumor and viral antigens to T lymphocytes), and increase their endocytosis and delivery to lysosomes (13–15).

Higher levels of *APLP2* mRNA are present in the pancreas after partial pancreatectomy, suggesting that APLP2 may have a function in regeneration of pancreas tissue (16). Furthermore, a few studies have shown increased expression of APLP2 in cancers. For example, in a screen of tumors, APLP2 was found to be overexpressed (17) and APLP2 was discovered to be elevated in invasive breast cancer adenocarcinoma compared to non-invasive adenocarcinoma (18). Among the many cancer cell lines that we previously examined, APLP2 was expressed at the highest level in the pancreatic cancer cell lines SUIT-2 and a SUIT-2 subline, S2-013 (19). Regulated intramembrane proteolysis is a process by which APLP2 or APP C-terminal fragments are liberated from secreted, extracellular N-terminal fragments (1,20–23). This process has been particularly noted in the BxPC3 pancreatic cancer cell line, which has been reported to exhibit a high level of APP cleavage; however, the accompanying expression and cleavage of APLP2 in this cell line was not examined (24). Proteolysis of APLP2 or APP can be accomplished by the β-site APP cleaving enzyme 1 (BACE1) or BACE2 (22,23,25). In the context of Alzheimer’s disease, BACE1 and BACE2 cleavage of APP has been well characterized, and both conserved and unique cleavage sites on APP have been demonstrated for the two BACE proteins (26–28). Recently, one BACE1 cleavage site in APLP2 was identified (23); however, BACE2 cut site(s) in APLP2 remain(s) unknown. Both BACE proteins have been reported in pancreatic tissue, but reports differ on BACE1 and BACE2 expression and activity in pancreatic ductal and acinar cells (22,23,27,29–32), which are cell types proposed to give rise to pancreatic cancer (33).

In our current studies, we have identified increased APLP2 in human pancreatic cancer tissues, as compared to normal pancreatic tissues, and have investigated the forms of APLP2 expressed in pancreatic cancer cell lines. We observed high molecular mass APLP2, at the molecular mass previously shown to be modified by glycosaminoglycans (GAG) (20,34,35), in the majority of pancreatic cancer cell lines, as well as full-length APLP2 without GAG modification and 12–15 kDa C-terminal fragments generated from secretase cleavage (22,23) in all these cell lines. C-terminal fragments of APP were only abundantly observed in the BxPC3 cell line in our panel of pancreatic cancer cell lines, suggesting that cleavage of APLP2, rather than APP, is a consistent molecular feature of pancreatic cancer cell lines. Furthermore, we have shown that transformation of pancreatic ductal cells by transfected oncogenes induces an increase in APLP2 expression, with particular enhancement in the expression of the APLP2 C-terminal fragments. Downregulation of APLP2 and/or APP in the pancreatic cancer S2-013 cell line, which displays representatively low expression of APP C-terminal fragments, decreased cell proliferation, suggesting a role for both family members in the growth of pancreatic cancer cell lines. Finally, treatment with inhibitors of β-secretases, enzymes that cleave APLP2 or APP to release C-terminal fragments, decreased the growth and viability of the pancreatic cancer cell line S2-013 but not of a non-transformed pancreatic ductal cell line. Overall, these studies suggest that APLP2 undergoes extensive modification and cleavage in pancreatic cancer cell lines, APLP2 (and APP) facilitate pancreatic cancer cell growth, and treatments that block APLP2 cleavage can diminish the growth of pancreatic cancer cells.

## Materials and methods

### Antibodies and immunostaining

Rabbit polyclonal antibodies against the full-length form of APLP2, the APLP2 C-terminus and the APP C-terminus were purchased from EMD Biosciences (San Diego, CA, USA). Mouse monoclonal anti-actin antibody was purchased from Novus Biologicals (Littleton, CO, USA). The secondary antibodies used for western blot analysis were peroxidase-conjugated AffiniPure goat anti-mouse IgG light chain or peroxidase-conjugated IgG fraction mouse anti-rabbit IgG light chain (Jackson ImmunoResearch Laboratories, West Grove, PA, USA).

Tissue sections were obtained from the University of Nebraska Medical Center (UNMC) Rapid Autopsy Program, according to a protocol approved by the UNMC Institutional Review Board. All tissue donors had provided written consent. For immunostaining, paraffin-fixed sections were stained with anti-APLP2 antibody before evaluation in a blinded manner, with scoring for APLP2 expression (− for negative; weak for low expression; + for moderate expression; ++ for strong expression).

### Cell lines and culturing conditions

The pancreatic cancer cell lines used in these studies were BxPC3, Capan-2, Hs766T, SUIT-2 and S2-013 (36–41). The S2-013 cell line is a cloned subline of the SUIT-2 human pancreatic tumor cell line (which was derived from a liver metastasis) (37,39). The hTERT-HPNE cell line is a line of telomerase-immortalized cells from normal human pancreatic ducts. This cell line lacks cancer-associated changes, has a normal karyotype, and can serve as the progenitor of pancreatic ductal cells (42,43). The hTERT-HPNE cell line and transfectants have been previously used in studies of pancreatic ductal cell transformation (44–46).

All the human pancreatic cancer cell lines used in these studies were cultured in Roswell Park Memorial Institute (RPMI)-1640 medium that was supplemented with 10 or 15% (vol/vol) fetal bovine serum, 1 mM sodium pyruvate, 2 mM L-glutamine, 100 U/ml penicillin and 100 μg/ml streptomycin. The hTERT-HPNE cells were grown in Medium D (as described in reference 43) or in Dulbecco’s modified Eagle’s medium supplemented with 10% v/v fetal bovine serum, 1 mM sodium pyruvate, 2 mM L-glutamine, 100 U/ml penicillin and 100 μg/ml streptomycin. Basal media and additives were purchased from Invitrogen (Carlsbad, CA, USA) and fetal bovine serum was obtained from Atlanta Biologicals (Lawrenceville, GA, USA).

Downregulation of target proteins in culture was achieved by transient transfections of short interfering RNA (siRNA). ON-TARGETplus SMARTpool siRNA against human APLP2 or APP was obtained from Thermo Scientific Dharmacon (Lafayette, CO, USA). ON-TARGETplus control, non-targeting pool was used as a negative control (Thermo Scientific Dharmacon). Transfections were performed following the manufacturer’s instructions for cells in base maintenance medium. Briefly, cells were seeded at 1×10^5^ cells/well in a 6-well plate the day before transfection and the medium was exchanged on the day of transfection. DharmaFECT transfection reagent no. 1 (Thermo Scientific Dharmacon) was incubated with 0.4 pmol of siRNA for 20 min at room temperature and the mixture was added drop-wise to wells. Reduced expression of target proteins was confirmed by western blot analysis of cell lysates.

### Preparation of cell lysates

Cells (1×10^7^) were harvested, washed three times in 20 mM iodoacetamide (with centrifugation for 5 min at 450 x g each time), and resuspended in cell lysis buffer (150 mM NaCl, 5 mM EDTA, 20 mM Tris-HCl pH 7.5, 0.5% Triton X-100). Resuspended cells were incubated on ice for 1 h with occasional mixing, and then stored at −80°C overnight. The following day, the cell lysates were thawed on ice, and then centrifuged at top speed in a desktop microcentrifuge for 30 min at 4°C. The supernatants were transferred to new tubes and stored at −80°C. Aliquots of lysates were mixed with 5X sodium dodecyl sulfate loading dye (250 mM Tris-HCl pH 6.8, 10% w/v sodium dodecyl sulfate, 30% v/v glycerol, 5% v/v β-mercaptoethanol, 0.02% w/v bromophenol blue) and boiled for 5 min prior to gel loading.

### Western blot analysis

Cell lysate samples were boiled for 5 min and then loaded on 4–20% Tris-glycine pre-cast gels (Invitrogen). Electrophoresis was performed at 125 V at room temperature. The proteins were transferred at 30 V for 2 h at room temperature to polyvinylidene difluoride Immobilon-P membranes (Millipore, Billerica, MA, USA). After overnight blocking in a 5% w/v solution of nonfat dry milk, the membranes were incubated with primary antibodies (diluted with 5% nonfat dry milk). Incubation with anti-actin antibody (1:2,000) was for 1 h and incubation with anti-full-length APLP2 antibody (1:1,000), anti-APLP2 C-terminus antibody (1:1,000) or anti-APP C-terminus antibody (1:1,000) was for 1.5 h. After primary antibody incubation, 3 washes with 0.05% Tween-20 in phosphate-buffered saline (PBS) for 10 min were performed. The membranes were subsequently incubated for 1 h in secondary antibodies, diluted 1:10,000 in 0.05% Tween-20 in PBS, and washed 3 times for 10 min with 0.3% Tween-20 in PBS. For protein visualization, the membranes were incubated for 1 min in Pierce ECL Western Blotting Substrate, or (for anti-APLP2 C-terminus) in Pierce SuperSignal West Pico Chemiluminescent Substrate (Thermo Scientific, Rockford, IL, USA) and exposed to Kodak BioMax MR film (Rochester, NY, USA). For densitometry of protein bands, quantification was done with the Molecular Imager ChemiDoc XRS system with Quantity One 1-D analysis software (BioRad, Hercules, CA, USA).

### Assessment of cellular growth

Growth of cells was assessed using thiazol blue (3-[4,5-dimethylthiazol-2-yl]-2,5-diphenyltetrazolium bromide, MTT) purchased from Sigma (St. Louis, MO, USA), by the MTT assay. Briefly, cells were seeded in 6-well plates at 1×10^5^ cells per well 24 h prior to the start of any cell culture treatments. The addition of β-secretase inhibitors to S2-013 or hTERT-HPNE cells signified the start of the time course. For siRNA studies, S2-013 cells were transfected with siRNA for 48 h to reduce expression of the target protein and re-seeded at 5×10^4^ cells per well, which then served as the start of the MTT time course. At the time point indicated, culture medium was removed and cells were incubated in 2.5 ml/well of 1 mg/ml MTT reagent (dissolved in base maintenance medium lacking phenol red) for 3 h at 37°C in 5% CO_2_. Suspension cells were collected pre- and post-incubation in MTT by centrifugation. The MTT solution was then removed and the metabolized MTT reagent was dissolved in isopropanol. Aliquots of the isopropanol-MTT solution were transferred a 96-well microtiter plate in replicate, and absorbances at 570 nm and 690 nm were taken on a SpectraMax M5e instrument (kindly provided by Dr Amar Natarajan, University of Nebraska Medical Center) using SoftMax Pro software (Molecular Devices, Sunnyvale, CA, USA). To determine MTT-specific absorbance, A690 was subtracted from A570.

### β-secretase inhibition experiments

The cells were seeded at low density in 6-well plates and allowed to adhere for 24 h prior to treatment with β-secretase inhibitors (signifying 0 h). The inhibitors used were NB2-755, −281, −418, −897 (donated by Novartis, Basel, Switzerland), or β-secretase inhibitor IV (purchased from Calbiochem/EMD Biosciences). The inhibitors were added at 2 μM and untreated wells were used as controls. Cells were assayed at 0, 24, 48 and 72 h by the MTT assay (described above) or by mixing aliquots of the cells with 0.4% trypan blue stain (Invitrogen) and counting the cells on a hemocytometer. The percentage of viable cells was calculated as unstained cell number divided by total cell number x 100. At the 24 h time point, samples of S2-013 cells treated with the Novartis inhibitors were also collected for preparation of cell lysates and western blot analysis (described above).

### Statistical analysis of data

To determine statistical differences in results obtained in the MTT growth assay, the analysis of variance (ANOVA) test was applied with the criterion for significance set at p<0.05. Statistical differences in the results from experiments utilizing the Calbiochem β-secretase inhibitor were determined by the Student’s t-test and the criterion for significance was set at p<0.05.

## Results

### APLP2 is expressed, modified by GAG, and cleaved in human pancreatic cancer cell lines

**Figure 1:**
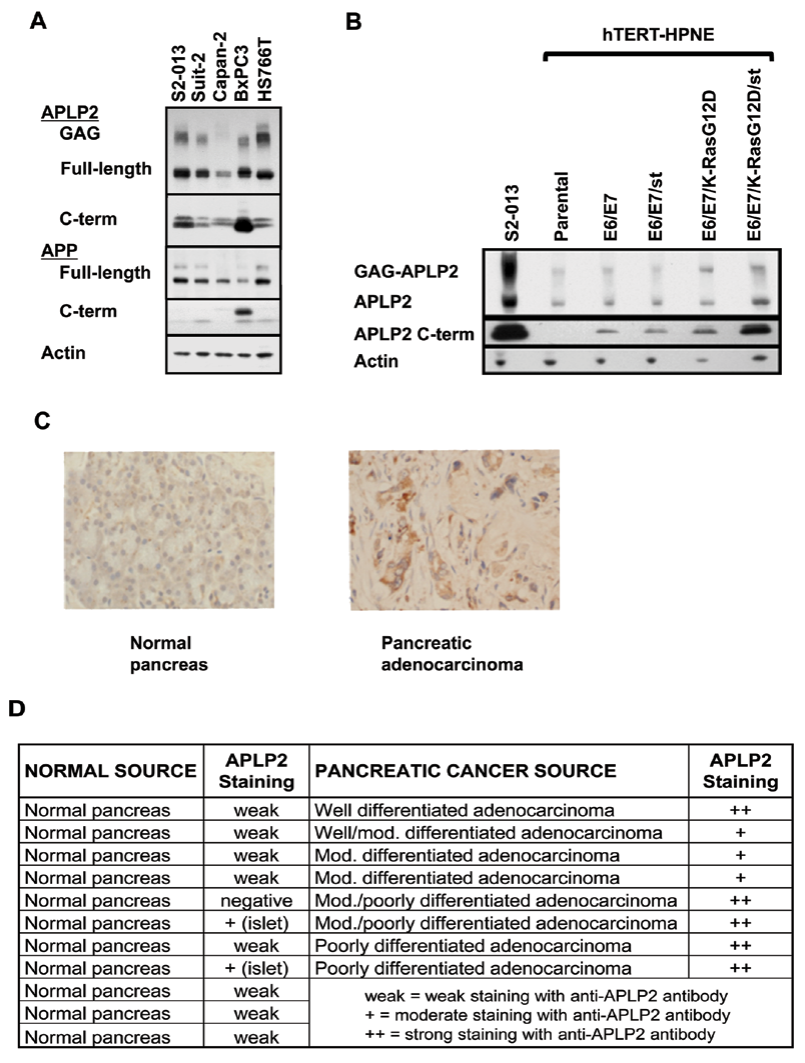
APLP2 expression and post-translational modification are elevated in pancreatic cancer. (A) GAG-modified APLP2, full-length APLP2 and APLP2 C-terminal fragments were found to be highly expressed in several human pancreatic cancer cell lines by western blot analysis of cell lysates. Antiserum raised against full-length APLP2, the C-terminus of APLP2 (top panel), or the C-terminus of APP (middle panels) were used to detect protein fragments based on molecular mass. The full-length form was identified at ∼100 kDa, glycosaminoglycan (GAG)-modified form around 250 kDa (APLP2 only) and C-terminal fragments between 10–15 kDa. Anti-actin (bottom panel) was used as a loading control. (B) Western blots for APLP2 expression in hTERT-HPNE (pancreatic ductal cells expressing telomerase, indicated as parental), with the stable addition of oncogenes: human papilloma virus genes E6 and E7 (E6/E7), SV40 small t antigen (st) and/or mutant K-Ras (K-RasG12D) (44,45). Addition of all oncogenes to hTERT-HPNE results in transformation of the cell line. The S2-013 pancreatic cancer cell line was used as a positive control for APLP2 and actin was used as a loading control. (C) Representative immunostaining images for APLP2 (brown) in normal pancreatic tissue and pancreatic adenocarcinoma tissue (×400 magnification). Formalin-fixed and paraffin-embedded tissue sections obtained from the UNMC Pancreatic Cancer Rapid Autopsy Program were stained with anti-APLP2 antibody before evaluation in a blinded manner. Cell nuclei (blue) were counter-stained by Mayer’s hematoxylin. (D) Summary of scoring for anti-APLP2 staining of a series of normal pancreas and pancreatic adenocarcinoma tissue samples. Overall, pancreatic adenocarcinoma tissue showed higher expression of APLP2 compared to normal pancreas tissue.

Previously, we found high levels of APLP2 expression in several cancer cell lines, including pancreatic cancer lines (S2-013, SUIT2 and Hs766T), prostate cancer lines and a melanoma line (19). We have confirmed the high expression of APLP2 in pancreatic cancer cell lines, using additional pancreatic cancer lines (BxPC3 and Capan-2) and observed additional bands in the molecular mass range previously shown to be GAG-modified APLP2 (20,34,35; Fig. 1A). Notably, earlier studies by others (using corneal epithelium and Chinese hamster ovary cells) have shown that expression of GAG-modified APLP2 correlates with increased cellular migration (7,8). Thus, the extensive GAG modification of APLP2 in pancreatic cancer cells may have a pro-migratory influence.

In addition to APLP2, APP was expressed in each of the pancreatic cancer cell lines (Fig. 1A). The two bands of APP represent immature (bottom) and mature (upper) full-length protein, with the higher molecular mass of the mature form due to glycosylation (47). APP has been shown to promote proliferation of the BxPC3 pancreatic cancer cell line, conferring this activity through the soluble N-terminal fragment (24,48), and soluble APLP2 has been identified in the secretome of another pancreatic cancer cell line, SUIT-2 (49). Liberation of soluble APP and soluble APLP2 occurs following secretase cleavage of the full-length proteins, simultaneously creating 12–15 kDa intracellular C-terminal fragments (1,20–23). We determined the expression of C-terminal fragments for APP and APLP2 in our panel of pancreatic cancer cell lines by western blot analysis. APLP2 C-terminal fragments were present in all cell lines examined, whereas APP C-terminal fragments were only substantially present in the BxPC3 cell line (Fig. 1A). In some cell types, APLP2 or APP C-terminal fragments have been shown to be additionally be cleaved by γ-secretase, generating smaller, 4 kDa C-terminal fragments (1,2,20). These smaller fragments of APLP2 or APP were not observed in our panel of pancreatic cancer cell lines, even when samples were electrophoresed on 18% Tris-glycine gels and the film was exposed overnight (data not shown). These data implicate APLP2 as the primary cleavage target of secretases in the majority of pancreatic cancer cells, rather than APP. We therefore pursued the function of APLP2 expression and cleavage in transformed pancreatic cells.

### Oncogene expression increases the presence of APLP2 and of APLP2 C-terminal fragments

Our identification of GAG-modified APLP2 and APLP2 C-terminal fragment expression in pancreatic cancer cell lines did not clarify whether these forms of APLP2 occur only in the transformed state, or if GAG-APLP2 and APLP2 C-terminal fragments are also endogenously present in untransformed pancreatic ductal cells. To address this question, we determined the expression of APLP2 in a series of cell lines generated from the hTERTHPNE cell line, which has been well characterized and used previously in several pancreatic cancer studies (42–46). The hTERT-HPNE cell line is an immortalized, but non-transformed, cell line derived from human pancreatic ductal epithelium (42,43), and with oncogene-transfected derivatives of this line it has been demonstrated that expression of the human papillomavirus E6 and E7 oncogenes (which block p53 and Rb function, respectively) and SV40 small t antigen (st) can permit mutant K-Ras transformation (44,45). With these cell lines we have been able to compare expression of full-length APLP2, GAG-modified APLP2 and APLP2 C-terminal fragments in transformed versus matched, non-transformed pancreatic cells. By western blot analysis, we demonstrated that all APLP2 forms examined are over-expressed in the transformed hTERT-HPNE E6/E7/K-RasG12D/st cells, compared to the related, untransformed cell lines (Fig. 1B). The pancreatic cancer cell line S2-013 was used as a positive control for APLP2 forms observed in pancreatic cancer cell lines. The most dramatic enhancement in APLP2 expression during oncogene-induced transformation of pancreatic ductal cells was detected in the APLP2 C-terminal cleavage fragments (Fig. 1B, middle panel). These data indicate that the presence of APLP2, and even more so the presence of APLP2 C-terminal fragments, is increased by oncogene expression in pancreatic ductal cells, and especially by oncogene-mediated pancreatic ductal cell transformation.

### Expression of APLP2 in human pancreatic cancer tissue

By immunostaining, we also examined whether APLP2 expression was increased in human pancreatic cancer tissue, relative to normal tissues. As indicated in Fig. 1 (C and D), APLP2 was weakly expressed in 8 out of 11 normal pancreas samples, and not detected at all in 1 normal pancreas sample. In 2 other normal pancreas tissue samples, APLP2 was detected at a moderate level, but only in the islets. Higher APLP2 expression was found throughout all 8 pancreatic cancer samples: in 5 samples, APLP2 staining was detected at a strong level, and in 3 samples at a moderate level. Among the 5 samples with strong APLP2 staining, 4 were either moderate/poorly differentiated or poorly differentiated pancreatic adenocarcinoma, confirming our observation from the hTERT-HPNE cell lines that APLP2 expression may increase as pancreatic cancer cells diverge further from normal morphology.

### Loss of APLP2 and/or APP impairs the growth of S2-013 pancreatic cancer cells

**Figure 2:**
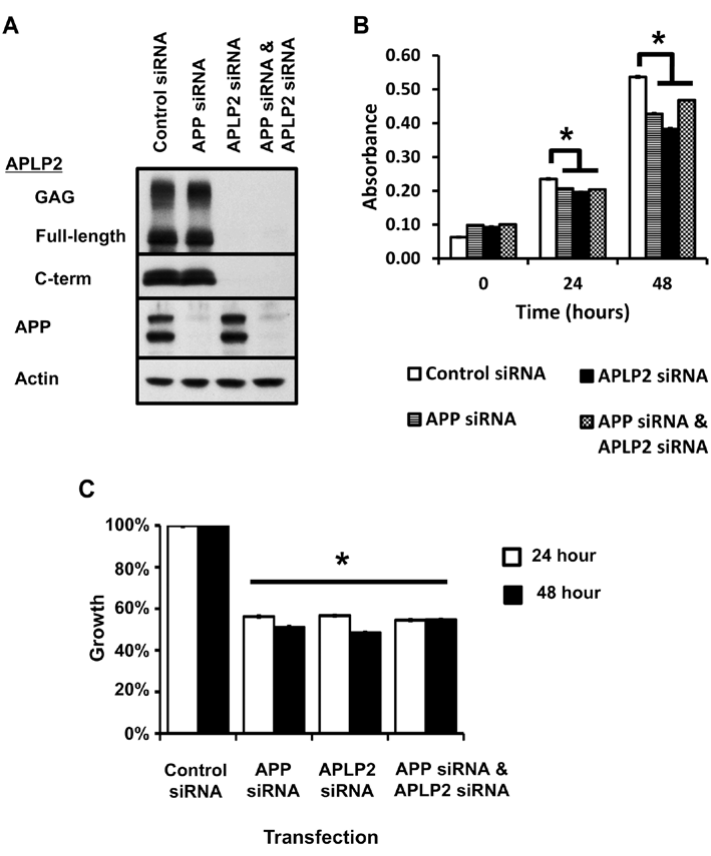
Reduction of APLP2 and/or APP by siRNA impairs the growth of a pancreatic cancer cell line. S2-013 cells were transfected with pooled siRNA against APP and APLP2, alone or in combination. Non-targeting pooled siRNA was used as the negative control (control siRNA). (A) Western blot analysis confirmed specific knockdown of glycosaminoglycan (GAG)-modified APLP2, full-length APLP2, APLP2 C-terminal fragments and/or full-length APP (mature, top band; immature, bottom band) actin 48 h post-transfection. Actin was used as a loading control. APP siRNA does not affect APLP2 expression, and vice versa. (B) S2-013 cells were seeded 48 h post-transfection at low density and cell growth was determined by the MTT assay. MTT-specific absorbance (y-axis) was obtained by subtracting the absorbance at 690 nm from the absorbance at 570 nm; time is displayed as hours post-seeding. (C) Reduction of APP and APLP2 significantly reduced the growth of S2-013 compared to cells treated with control siRNA. Percent inhibition in growth was obtained from the data in (B) by normalizing the MTT-specific absorbance to control siRNA absorbance and seeding at 0 h. Asterisks in (B and C) denote significance by the analysis of variance (ANOVA), p<0.001; n=12, error bars indicate standard error of the mean.

Downregulation of APP by siRNA has been shown to impair the growth of BxPC3 cells (11, 29), which we found have enhanced processing of APP compared to other pancreatic cancer cell lines (Fig. 1A). However, the contribution of APP to the growth of pancreatic cancer cells with minimal expression of APP C-terminal fragments has not been assessed previously. To determine the effect on such cells, the S2-013 cell line (which we identified as having relatively low APP C-terminal fragment expression, Fig. 1A) was transfected with siRNA against APP. Immature and mature (glycosylated) full-length APP (47) expression was reduced by 48-h post-transfection, without affecting APLP2 expression (Fig. 2A), and growth of cells was determined by the MTT assay. S2-013 cells transfected with APP siRNA demonstrated significantly reduced growth, compared to control siRNA (Fig. 2B and C), suggesting that full-length APP, rather than secretase cleavage of APP, contributes to the growth of pancreatic cancer cell lines.

Since we had observed increasing APLP2 expression with transformation of pancreatic cancer cells (Fig. 1), we then determined the contribution of APLP2 to the growth of S2-013 cells. Western blot analysis of S2-013 cells transfected with siRNA against APLP2 for 48 h revealed downregulation of all APLP2 forms: full-length APLP2, GAG-modified APLP2 and APLP2 C-terminal fragments (Fig. 2A). Growth of S2-013 cells was significantly impaired following transfection with siRNA against APLP2, compared to control (Fig. 2B and C). Downregulation of APLP2 had a growth-inhibitory effect on S2-013, despite maintained expression of APP (Fig. 2). Knockdown of APLP2 or APP comparably inhibited the growth of S2-013 cells (Fig. 2), demonstrating that loss of either protein had a deleterious effect on cell growth. We then reduced expression of APLP2 and APP by co-transfection with both siRNAs to determine if loss of both proteins would further restrict the growth of pancreatic cancer cells. Reductions in expression of all forms of APLP2 and APP at 48-h post-transfection were confirmed by western blot analysis (Fig. 2A). APLP2 siRNA and APP siRNA co-transfected S2-013 cells displayed an inhibition in growth compared to cells treated with control siRNA, but not compared to S2-013 cells that were transfected with either APLP2 siRNA or APP siRNA alone (Fig. 2B and C). These data demonstrate that both APLP2 and APP contribute to the growth of pancreatic cancer cells, and simultaneous loss of both proteins does not further enhance the growth inhibition of pancreatic cancer cells. Therefore, it is probable that APLP2 and APP act through the same pathway to promote the growth of pancreatic cancer cells. Because loss of one protein still reduced pancreatic cancer cell growth, we can conclude that the remaining protein cannot compensate for the loss of the other. These data suggest that APLP2 and APP have unique roles within the same pathway.

### Blocking β-secretase activity reduced pancreatic cancer cell viability

**Figure 3:**
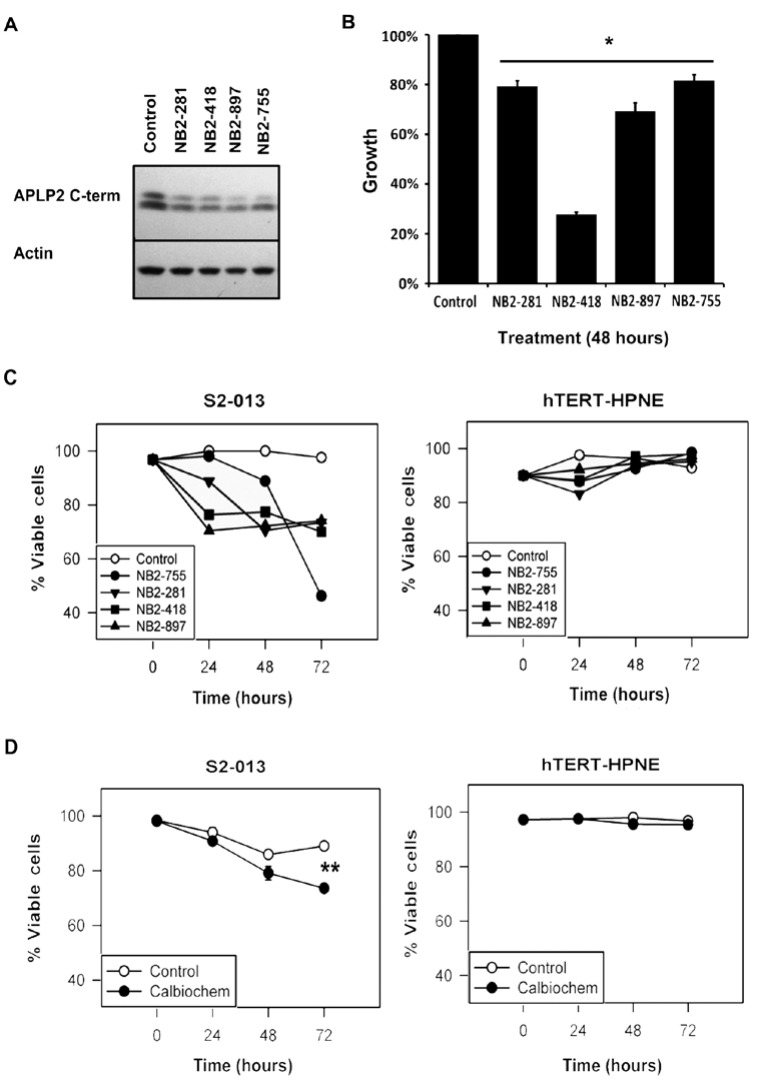
Chemical inhibition of β-secretase reduced APLP2 C-terminal fragment expression and pancreatic cancer cell viability. (A) Western blot analysis showed reduced expression of APLP2 C-terminal fragments in S2-013 cell lysates following 24-h incubation with 2 µM of each of four Novartis β-secretase inhibitors (NB2-755, −281, −418 and −897). Mock-treated cells and actin were respectively used as controls for β-secretase inhibitors and loading. (B) Novartis β-secretase inhibitors impaired the growth of S2-013 cells. Cell growth was determined by the MTT assay and values were compared to respective control cells, which were set as 100% (asterisk denotes p<0.005 by the Student’s t-test; n=6, error bars indicate standard error of the mean). (C) The viability of S2-013 cells in culture was reduced over time by β-secretase inhibition, as demonstrated by trypan blue staining following treatment with 2 µM NB2-755, −281, −418 or −897. In contrast, the viability of the hTERT-HPNE pancreatic ductal cells was not adversely affected by the same treatments with β-secretase inhibitors. Viability is expressed on the graph as the percentage of live cells, with the 0 h time point for control cells set at 100%. (D) S2-013 cells treated with 2 µM Calbiochem β-secretase inhibitor also exhibited impaired viability. For S2-013, the asterisk denotes p<0.001 at 72 h by the Student’s t-test. The viability of the control hTERT-HPNE cells was not reduced by incubation of the cells with the Calbiochem β-secretase inhibitor (p<0.05 by the Student’s t-test). The data shown are representative of the findings from two separate experiments, each with n=3 and the error bars represent the standard error of the mean.

The data shown in Fig. 1B suggest a relationship between oncogene expression and the cleavage of APLP2, and APLP2 C-terminal fragments were observed in all pancreatic cancer cell lines examined (Fig. 1A). In order to test the impact of blocking APLP2 cleavage on the growth of pancreatic cancer cells, we incubated S2-013 cells with chemical inhibitors of the β-secretase enzymes, which subsequently reduced the production of APLP2 C-terminal fragments within 24 h (Fig. 3A). By the MTT assay, we observed that the presence of a β-secretase inhibitor impaired the growth of S2-013 cells compared to mock-treated cells (Fig. 3B). The reduced growth of S2-013 was accompanied by a reduction in the number of viable cells over time (Fig. 3C and D). In contrast, culturing the non-transformed hTERT-HPNE pancreatic cells with the β-secretase inhibitors did not influence the growth and survival of the cells (Fig. 3C and D). Although β-secretases are known to cleave molecules in addition to APLP2 (50–52), and cleavage of other proteins may contribute to the effect of β-secretase inhibitors on pancreatic cancer cell viability, these results are consistent with the notion that APLP2 cleavage fragments influence the survival of pancreatic cancer cells, and they suggest that further investigation of the efficacy and mechanism of β-secretase inhibitors as potential therapies for pancreatic cancer is warranted.

## Discussion

APLP2, like APP, has been implicated in Alzheimer’s disease, although APLP2’s sequence does not include the β-amyloid peptide found within APP (1,53). In research to develop improved treatments for Alzheimer’s disease, there has been considerable investigation of the cleavage of APP and APLP2, and inhibitors of β-secretases have been developed that block the cleavage of these proteins (1,54–56). Two β-secretases, β-site APP-cleaving enzyme (BACE)1 and BACE2, have been implicated in the cleavage of APP family members (50,56). BACE2 is expressed in the pancreas (30) and specifically in pancreatic ductal cells (31), although other reports place BACE2 expression within islet cells (32). The relative contribution of BACE1 and BACE2 to the overall activity of β-secretase in pancreatic cancer cell lines and tissues remains an area of active research. The results shown in Fig. 3 include data derived with four β-secretase inhibitors produced by Novartis (NB2-755, −281, −418 and −897) and one from Calbiochem, all with activity towards BACE1 and BACE2. The commercially available Calbiochem β-secretase inhibitor has 15 times greater ability to inhibit BACE1 compared to BACE2 (according to the manufacturer) and it required prolonged time in culture to inhibit pancreatic cancer cell survival (Fig. 3D). Ultimately, increased understanding of BACE1 and BACE2 expression and activity in pancreatic cancer will allow optimal selection of β-secretase inhibitors that have greater efficacy in reducing the viability of pancreatic cancer cells.

APLP2 and APP are capable of influencing growth signaling pathways through phosphorylation, subcellular localization and interactions with protein binding partners, which are in turn regulated by secretase cleavage and post-translational modifications (1,2,20,21). In order to interpret Fig. 2 and to clarify the pathway relationship of APLP2 and APP in pancreatic cancer cell proliferation, Table I was constructed exploring various pathway relationships between APLP2 and APP. The anticipated outcome on cell proliferation following downregulation of APLP2 and/or APP was used as the functional readout (fourth and fifth columns, Table I). Three basic questions were considered for the construction of the table. First, are APLP2 and APP in the same pathway? If APLP2 and APP are not in the same pathway, parallel pathways and unrelated pathways should be considered. As shown in Fig. 2, simultaneous downregulation of both APLP2 and APP did not enhance growth inhibition beyond down-regulation of APLP2 or APP alone, signifying that APLP2 and APP likely act upon the same pathway in pancreatic cancer cell growth (eliminating the scenarios in the bottom half of Table I). Second, what is the relationship between APLP2 and APP within the pathway? In the same pathway, APLP2 and APP could act at the same point, or sequentially to one another, or in a parallel (or switchboard) relationship. The last question considered was whether APP and APLP2 have the same role. If APLP2 and APP cannot exchange for one another, they evidently have unique roles. Because growth inhibition was still observed when either APLP2 or APP was downregulated, APLP2 expression does not compensate for loss of APP in pancreatic cancer cell growth, and vice versa. Therefore, pathway relationships outlined in rows 1 and 4 from Table I do not match the experimental data (Fig. 2). Consequently, two models were devised (rows 2 and 3) that match the experimental data from Fig. 2.

The second row of Table I indicates that APLP2 and APP could be involved at the same point within the same pathway, but yet serve unique roles; an example of this scenario would be a functional heterodimer of APLP2 and APP. Heterodimers of APLP2 and APP are known to form (57). In this context, a homodimer of APLP2 or APP could not recapitulate the function of the APLP2-APP heterodimer, and equal growth inhibition would occur with loss of one or both proteins. The scenario in the third row also matches our experimental data. Instead of APLP2 or APP acting at the same point in a pathway, one protein would be upstream of the other, and both would be required for a growth signal to be conducted. In both devised scenarios matching the experimental data, APLP2 and APP would have unique roles in the growth of pancreatic cancer cells. Ultimately, the conclusion best fitting the data is that APLP2 and APP serve unique roles in the growth of pancreatic cancer cell lines.

Downregulation of APLP2 C-terminal fragments (by siRNA or β-secretase inhibitors) resulted in growth inhibition of S2-013 cells, despite maintained expression of APP full-length protein (Fig. 2A and B and data not shown); this is noteworthy since β-secretase inhibitors are also capable of reducing cleavage of APP. However, APP appears to be a poor target for secretases expressed in pancreatic cancer cell lines, as evidenced by minimal expression of APP C-terminal fragments in the majority of pancreatic cancer cell lines (Fig. 1A) and failure of APLP2 reduction (by siRNA or β-secretase inhibitors) to enhance APP C-terminal cleavage (data not shown). Selective cleavage of APLP2 over APP can arise through secretase specificity, intracellular compartmentalization, APLP2-APP homo- or hetero-oligomerization and/or isoforms of APLP2 or APP expressed (58–63). Full-length APLP2 and APP are capable of forming homo- or hetero-oligomers, which may be disrupted upon soluble APLP2 or soluble APP binding (57,65–67). The transmembrane-soluble complexes formed by APLP2 and APP are significant because the activity of APLP2 and APP receptors depends upon the complex formed. For example, dimers of full-length APP have been proposed to activate cell death in neuronal cells, where full-length APP-soluble APP dimers disrupt the cytotoxic signal (67). While in our experiments protein expression of APLP2 or APP was not altered following loss of the other family member, alterations to additional regulatory mechanisms of APLP2 and/or APP may occur. Future investigations are required to explore the possibilities of altered APLP2 or APP regulatory mechanisms and to dissect functional identities of APLP2 and APP in pancreatic cancer growth and viability.

Treatment with β-secretase inhibitors caused not only a decrease in APLP2 C-terminal fragments, but also a reduction in S2-013 cell viability (Fig. 3). Our data indicate that inhibitory therapies that target APLP2, APP and BACE may be promising for the treatment of pancreatic cancer. Notably, tolfenamic acid, which is currently under investigation for use in pancreatic cancer (68–71), has been shown to impair expression of APP and BACE (72). Reduced cleavage of other proteins (in addition to APLP2) might also contribute to the ability of the β-secretase inhibitors to affect pancreatic cancer cell viability. The β-secretase inhibitors target β-secretases that cleave APP as well as APLP2. However, our analysis has not shown a very high expression of APP C-terminal fragments in pancreatic cancer cell lines except BxPC3 (Fig. 1). Cleavage of additional proteins (not in the APP/APLP2 family) able to influence viability may be affected by the β-secretase inhibitors. BACE2 substrate proteins are not well defined, although there is somewhat more information on BACE1 substrates, which include heregulins (73–75). Heregulin proteins have been noted to be over-expressed in pancreatic cancer cells and to influence their growth (76). Thus, heregulins might have a role in β-secretase inhibitor-mediated reduction of pancreatic cancer cell viability. Additional credence for β-secretase inhibitors as a therapy for pancreatic cancer could be obtained by conducting future studies to test β-secretase inhibitors in xenograft models of pancreatic cancer, alone or in combination with gemcitabine (used in clinical treatment for pancreatic cancer patients) (77).

The available treatments for pancreatic cancer are unable to decrease morbidity and mortality effectively, and only by attaining a better understanding of pancreatic cancer cell biology and testing novel therapies can we hope to increase the odds for patients with this disease. In this study, our findings have revealed several new aspects of the relationship between APLP2 and pancreatic cancer. First, APLP2 is highly expressed in human pancreatic cancer cell lines and clinical tissue samples, relative to normal pancreatic tissues. Second, C-terminal fragments are more consistently generated from APLP2 rather than APP in pancreatic cancer cell lines. Third, the presence of APLP2 and APLP2 C-terminal fragments is increased by pancreatic ductal cell transformation. Fourth, down-regulation of APLP2 and/or APP impairs the growth of a pancreatic cancer cell line, and each protein likely contributes to the same growth pathway, but in a unique manner. Finally, inhibitors of β-secretase reduce APLP2 cleavage and pancreatic cancer cell viability. Overall, our results suggest that β-secretase, APLP2 (and notably its C-terminal cleavage fragments), and APP influence the survival of pancreatic cancer cells.

## Figures and Tables

**Table I:** Phenotypic outcomes of postulated pathways including APP and/or APLP2.

Pathway type	APP and APLP2 pathway relationship	APP and APLP2 role	Growth of cells expressing APP siRNA or APLP2 siRNA	Growth of cells expressing APP siRNA and APLP2 siRNA
Same	Same	Same	No effect	↓
Same	Same	Unique	↓	↓ (to same extent as cells expressing either siRNA)
Same	Sequential	Unique	↓	↓ (to same extent as cells expressing either siRNA)
Same	Parallel	Unique	No effect	↓
Parallel	+ and +	Unique	↓	↓
Parallel	+ and inhibits inhibitor	Unique	↓	↓
Parallel	Inhibits inhibitor and inhibits inhibitor	Unique	↓	↓
Unrelated	Separate	Unique	↓	↓

↓ Denotes reduced cell growth;

+ signifies positively regulates cell growth.

## Abbreviations:

APLP2: amyloid precursor-like protein 2;
APP: amyloid precursor protein;
DMSO: dimethyl sulfoxide;
MTT: 3-[4,5-dimethylthiazol-2-yl]-2,5-diphenyltetrazolium bromide;
PBS: phosphate-buffered saline;
siRNA: short interfering RNA
